# Development and Validation of a Simple Method to Quantify Contents of Phospholipids in Krill Oil by Fourier-Transform Infrared Spectroscopy

**DOI:** 10.3390/foods11010041

**Published:** 2021-12-24

**Authors:** Se-Eun Park, Hyo-Yeon Yu, Sangdoo Ahn

**Affiliations:** Department of Chemistry, Chung-Ang University, Seoul 06974, Korea; seeun6472@naver.com (S.-E.P.); hyoyeonyu@gmail.com (H.-Y.Y.)

**Keywords:** krill oil, Fourier-transform infrared spectroscopy, second-derivative spectrum, phospholipids, phosphatidylcholine

## Abstract

This study focuses on developing a quantification method for phosphatidylcholine (PC) and total phospholipid (PL) in krill oil using Fourier-transform infrared (FT-IR) spectroscopy. Signals derived from the choline and phosphate groups were selected as indicator variables for determining PC and total PL content; calibration curves with a correlation coefficient of >0.988 were constructed with calibration samples prepared by mixing krill oil raw material and fish oil in different ratios. The limit of detection (LOD, 0.35–3.29%) of the method was suitable for the designed assay with good accuracy (97.90–100.33%). The relative standard deviations for repeatability (0.90–2.31%) were acceptable. Therefore, both the methods using absorbance and that using second-derivative were confirmed to be suitable for quantitative analysis. When applying this method to test samples, including supplements, the PC content and total PL content were in good agreement with an average difference of 2–3% compared to the ^31^P NMR method. These results confirmed that the FT-IR method can be used as a convenient and rapid alternative to the ^31^P NMR method for quantifying PLs in krill oil.

## 1. Introduction

Omega-3 polyunsaturated fatty acids play an important role in human physiology; therefore, they are widely used as health supplements [1,2,3]. Recently, krill oil extracted from Antarctic krill (*Euphausia Superba*) has attracted attention as a source of long-chain omega-3 fatty acids, eicosapentaenoic acid (EPA), and docosahexaenoic acid (DHA) [4]. Unlike in other marine fish oils, most EPA and DHA of krill oil exist in the form of phospholipids (PLs) rather than in the form of triglycerides (TGAs) [5,6,7,8]. The bioavailability of omega-3 fatty acids in krill oil is expected to be higher than that in other fish oils because PLs are relatively soluble in water [6]. The PL content of krill oil, which varies depending on various factors, such as species and age, usually ranges from 30% to 80% (*w*/*w*). It is also known that 60–96% (*w*/*w*) of the total PL is in the form of phosphatidylcholine (PC) and a relatively small amount of phosphatidylethanolamine (PE) is present [3,7]. Therefore, PL content and composition are important characteristics of krill oil.

The PL content of krill oil should be at least 30% (*w*/*w*) according to the Codex Alimentarius Commission (Codex) [9]; furthermore, phosphorous-31 nuclear magnetic resonance (^31^P NMR) spectroscopy is used as the official content analysis method [10]. ^31^P NMR spectroscopy is a very useful technique for quantifying PLs because it can simultaneously perform qualitative and quantitative analyses [11,12,13]. This is because the NMR signal is quantitative in nature, as it is directly proportional to the number of corresponding resonant nuclei [14]; however, modern high-field NMR spectrometers are rarely used as routine equipment in common laboratories for detecting ^31^P signals because they are expensive and require expensive maintenance, such as cryogenic cooling [15]. Chromatographic methods, such as thin-layer chromatography and high-performance liquid chromatography, have been used as routine techniques in the analysis of the PL composition of foods because they can also perform qualitative and quantitative analyses and are not as expensive to operate in a laboratory [16,17]. However, they have limitations in that it takes huge time and effort to develop an analytical method and requires a large amount of solvent and individual standards for all analytes.

Recently, Fourier-transform infrared (FT-IR) spectroscopy has been widely used as a rapid food analysis method due to its simple preparation and fast measurement [18]. Particularly, FT-IR spectroscopy is a simple yet effective technique in the field related to the authenticity and quality control of edible fats and oils [19,20,21,22,23,24,25]. For example, a method is officially used to determine the trans-fat content in edible oils and fats by FT-IR signals of trans-alkene double bonds [26]. Additionally, second-derivative FT-IR spectroscopy was used to discriminate the authenticity of perilla oil [27]. Very recently, a study was conducted to analyze the composition of EPA, DHA, and astaxanthin in krill oil by combining FT-IR and Raman spectroscopy [1].

Several studies have also reported on the quantification of PLs in foods using FT-IR spectroscopy [28,29,30]. Proctor et al. showed that the FT-IR method is applicable to PL quantitative analysis by comparing it with the phosphorus content analysis method to quantify the PL of soy lecithin [28,29,30]. However, they generated a calibration curve by diluting a PL standard in chloroform solvent, which was used for quantitative analysis of the diluted PL samples in the same way. Therefore, its application to real samples such as krill oil having complex matrix structures is limited.

The goal of the present study was to develop and validate a simple and rapid quantification method to determine the total PL content and PC content in krill oil samples using FT-IR spectroscopy as an alternative to the ^31^P NMR method. The second-derivative FT-IR spectrum was also used to enhance the apparent resolution of overlapping absorbance peaks and improve their quantification. Furthermore, the results were compared with those of the absorption spectrum. Calibration curves for quantitative analysis were prepared using samples in which krill oil raw materials and fish oil were mixed. The proposed FT-IR method was validated in terms of specificity, linearity, accuracy, precision, the limit of detection (LOD), and the limit of quantification (LOQ). Additionally, the developed method was applied to analyze various krill oil test samples; the results were compared with those of ^31^P NMR analysis to verify the applicability and appropriateness of the proposed method.

## 2. Materials and Methods

### 2.1. Materials

The United States Pharmacopeia (USP) reference standard of krill oil and European Pharmacopoeia reference standard PLs (PC and PE) were purchased from MERCK Korea (Seoul, Korea). The krill oil raw material used in producing supplements was acquired from Pulses (Gyeonggi-do, Korea). Additionally, seven krill oil supplements (soft capsules) were purchased from different retailers in Korea. Fish oil (from Menhaden, a crude source of omega-3 fatty acids) used to dilute krill oil samples was purchased from MERCK Korea. qNMR standard grade triphenyl phosphate (TPP) for a ^31^P NMR internal standard (ISD) was purchased from MERCK Korea. Most of the reagents were also purchased from MERCK: chloroform (HPLC grade), cesium carbonate (CsCO_3_, 99.9% trace metals basis), and ethylenedinitrilotetraacetic acid (EDTA, 99.995% trace metals basis). Chloroform-*d* (CDCl_3_, 99.8 atom% D, contains 0.03% (*v/v*) TMS) and methanol-*d*_4_ (≥99.8 atom% D) for ^31^P NMR measurement were purchased from BK Instruments (Daejeon, Korea).

### 2.2. Sample Preparation

Nine calibration standard samples were prepared in a range of 20 to 100 wt% of the krill oil raw material mixed with fish oil (PL free) in different ratios (20/80, 30/70, 40/60, 50/50, 60/40, 70/30, 80/20, 90/10, and 100/0 *w*/*w*) to generate the calibration curves to quantify PC and total PL content. The krill oil raw material was analyzed by the ^31^P NMR method specified in Codex to determine the PC and total PL content of each calibration sample [10]. As a result, they were found to be 49.86 and 56.55 wt%, respectively (Figure S1).

A total of 12 test samples were prepared, including five mixtures of the krill oil raw material and fish oil in arbitrary ratios and seven different krill oil supplement capsules, to verify the applicability of the developed FT-IR method. The supplement samples were diluted with fish oil in a 1:1 ratio and then used for analysis to ensure uniform measurement conditions.

### 2.3. FT-IR Spectroscopy

All spectra were measured by an FT-IR spectrometer (TENSOR-27; Bruker Optics GmbH, Karlsruhe, Germany) equipped with a diamond ATR system (A225/Q Platinum ATR; Bruker Optics GmbH). The spectra were obtained in absorbance units, in the wavelength range of 4000–400 cm^−1^, with 16 scans and a resolution of 4 cm^−1^. Measurements were repeated three times for each sample and averaged with OMNIC software (version 8.2, ThermoFisher Scientific Inc., Waltham, MA, USA) to ensure the reproducibility and representativeness of the obtained FT-IR spectra. Spectral data are often derivatized by the Savitzky-Golay (SG) numerical algorithm as a pre-processing step to resolve overlapping signals, enhance the significant spectral difference, and subdue unnecessary spectral characteristics caused by unusual equipment and sample properties. A second-derivative spectrum was calculated for each measured pixel using the SG numerical algorithm at the third-degree polynomial at seven points [31,32]. The derivative values were used for quantitative measurement and data processing using the peak height of the second-order spectra of the major spectral band.

### 2.4. Validation of Analytical Method

Validation of the proposed FT-IR method was performed according to the ICH guidelines. The specificity of the assay was confirmed by comparing the spectrum of a mixture of krill oil raw material and fish oil with those produced after adding PC and PE standards to that mixture. The linearity of the calibration curves prepared at nine different concentrations was evaluated by linear regression analysis. The accuracy was assessed with a spike and recovery method, which confirmed the recovery by spiking krill oil whose total PL content and PC content had been confirmed by the ^31^P NMR method, into a mixture of USP krill oil reference material with fish oil. Three repeated measurements were performed for three concentrations within the calibration curves for total PL and PC. The precision was assessed for repeatability and intermediate precision using the krill oil raw material. The repeatability was evaluated by six replicated measurements in same day, and the intermediate precision was determined by the variability of independent results obtained on three different days. The LOD and LOQ values for PC and total PL quantification were estimated using the equation based on the standard deviation of the response (σ) and the slope of the calibration curve (S): LOD = 3.3 × σ/S and LOQ = 10 × σ/S.

### 2.5. ^31^P NMR Spectroscopy

The quantitative analysis of PLs in krill oil samples by ^31^P NMR spectroscopy was conducted by referring to the USP monograph for krill oil [33] and previous studies [34]. Furthermore, 300–350 mg of krill oil and 20–25 mg of TPP (as an ISD) were precisely weighed and placed into a vial for measurements of ^31^P NMR; after that, 1 mL each of CDCl_3_, methanol-*d*_4_, and EDTA solution was added. The EDTA solution was prepared by dropping 1 M CsCO_3_ solution into 0.2 M EDTA and adjusting the PH to 7.2–7.5. The sample solution of krill oil was thoroughly mixed for 30 min and centrifuged to obtain the lower organic solvent layer in a 5-mm o.d. NMR tube. NMR measurements were performed using a 600-MHz NMR (242.9 MHz for ^31^P) spectrometer (VNS-600, Varian, Palo Alto, CA, USA). ^31^P NMR spectra were acquired by ^1^H decoupling with an inverse-gated pulse sequence for accurate quantitation. The NMR experimental conditions were as follows: 25 °C temperature, 45° pulse angle, 5.2 s acquisition time, and 128 scan averages with a 10 s relaxation delay. The chemical shifts of PL peaks were allocated relative to the TPP signal (−17.8 ppm). The qNMR equation (Equation (1)) was used for PC and total PL quantification from the ^31^P NMR spectrum [35].

(1) $$C_{PL}\left(wt\%\right)=\frac{I_{PL}}{I_{ISD}}\times \frac{MW_{PL}}{MW_{ISD}}\times \frac{w_{ISD}}{w_{sample}}\times P_{ISD}\times 100,$$

where *I*, *MW*, and *w* correspond to the integral value, molecular weight (g mol^−1^), and weight (g), respectively. *P_ISD_* and *C_PL_* denote the purity of *ISD* and content of the *PL* in terms of weight percentage (wt %), respectively.

## 3. Results and Discussion

### 3.1. FT-IR Spectrum of Krill Oil

**Figure 1:**
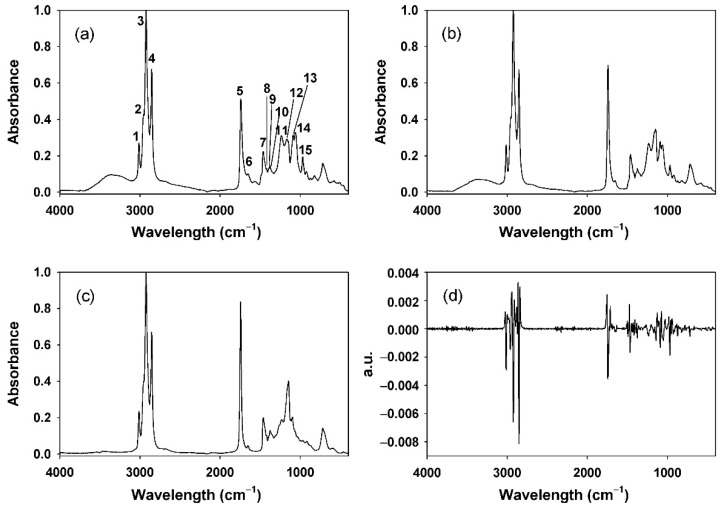
FT-IR absorption spectra of (**a**) USP reference standard krill oil sample, (**b**) fish oil, and (**c**) mixture sample of krill oil raw material and fish oil (50/50, *w*/*w*%). (**d**) Second-derivative FT-IR spectrum of (**c**). See Table 1 for each peak assignment.

The representative FT-IR spectra of krill oil and fish oil samples are shown in Figure 1 within the range of 4000–400 cm^−1^ [3,5]. Table 1 summarizes the assignment of significant FT-IR signals based on the previously reported literature [1,36,37].

In the FT-IR spectrum of the standard krill oil sample, characteristic signals derived from PL were identified along with typical fatty acid signals: symmetric/asymmetric phosphate diester stretch in PO_2_^−^ (1090 cm^−1^ and 1236 cm^−1^), symmetric/asymmetric ester stretch in –C–O–P (1060 cm^−1^ and 1167 cm^−1^), and asymmetric stretch in –N–(CH_3_)_3_ (970 cm^−1^) [36]. In the FT-IR spectrum of the fish oil sample (Figure 1b), although not derived from PL, vibration signals by various functional groups in other fatty acids also appeared in the region of 1100–1200 cm^−1^. Note that the fish oil used to control the concentration of krill oil samples did not contain PL, which was verified by ^31^P NMR spectroscopy (see Figure S2). The intensities of the absorption signals were normalized with that of the asymmetric stretching CH_3_ signal (I3, 2924 cm^−1^) [3,38,39]. The signal was suitable as a reference because it not only was the largest signal for both krill oil and fish oil samples, but also appeared almost constant regardless of the PL content.

### 3.2. Development of FT-IR Quantification Method

#### 3.2.1. Calibration Curves

**Figure 2:**
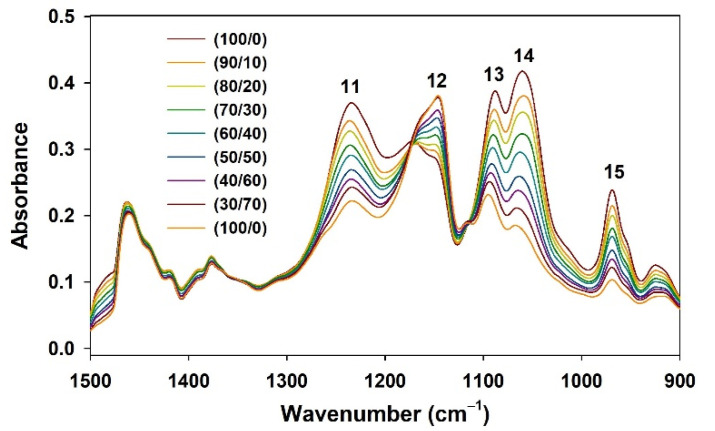
FT-IR spectra of mixed samples of krill oil raw material and fish oil with a ratio of krill oil/fish oil from 20/80 to 100/0 (*w*/*w*%) in the region of 1500–900 cm^−1^.

Among the signals derived from PL functional groups, the signals that were well separated and less disturbed by fish oil signals were carefully selected to determine the total PL content and PC content in krill oil samples: I11 and I15 for total PL and PC quantification, respectively. As indicated in Table 1, I11 is a signal related to the PO_2_^−^ group in the PL head, and I15 is a unique signal of the choline group in PC. Figure 2 shows that these two signals, which are relatively well separated from other signals, increase proportionally with increasing krill oil raw material ratio.

**Figure 3:**
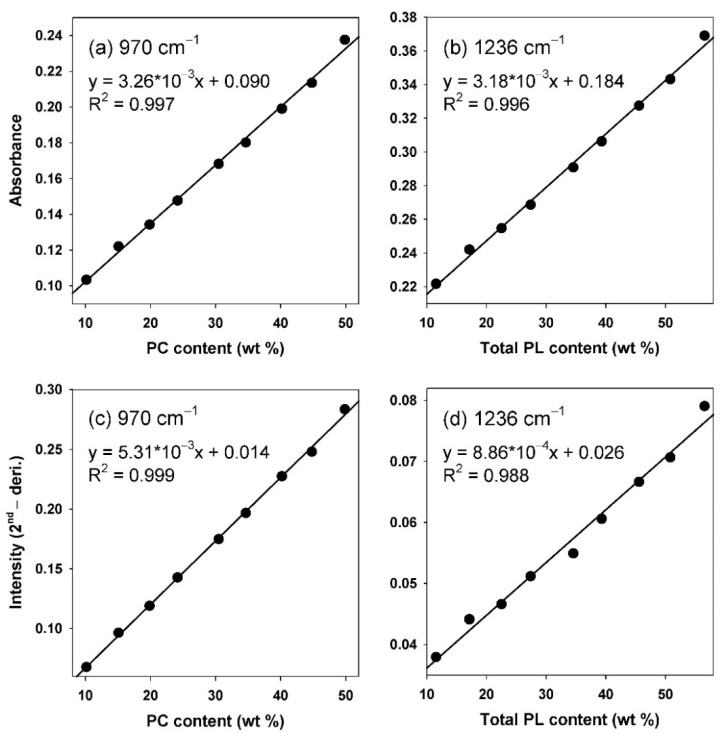
Calibration curves for PC and total PL content: absorbance and its second-derivative of (**a**,**c**) (CH_3_)_3_-N-asymmetric stretching (970 cm^−1^) signal and (**b**,**d**) -P=O asymmetric stretching (1236 cm^−1^) signal.

As shown in Figure 3, calibration curves for the PC and total PL content analyses in krill oil samples were constructed using two methods: using the absorbance of the indicator signals (I15 and I11) and using the second-derivative of these signals. The ranges of PC and total PL content in nine calibration samples corresponded to 10.16–49.86 wt% and 11.52–56.55 wt%, respectively.

The correlation coefficient (R^2^) of the calibration curves for the quantification of PC content (Figure 3a,c) showed a value greater than 0.99 in both the method—using absorbance and that using the second-derivative. This is because the -N-(CH_3_)_3_ asymmetric stretching signal (970 cm^−1^) used in the calibration curve is unique, derived from the choline group in PC, and very well separated from other signals; however, the correlation coefficient of the calibration curve for the total PL content in the absorbance method was 0.996 (Figure 3b), while it was 0.988—a relatively low value—in the method using the second-derivative (Figure 3d). This is considered because the characteristic signal for PL (PO_2_^−^ asymmetric stretching, 1236 cm^−1^) is broad; therefore, it becomes a small second-derivative peak (see Figure 3d) that can cause a relatively large error compared to the absorbance. It was confirmed that the error becomes larger when the calibration curve is constructed with PL characteristic signals (I12, I13, and I14) having a large degree of overlap with other signals; however, it was confirmed that both the absorbance and second-derivative methods are suitable for determining PC and total PL content in krill oil, considering the general experimental error level of FT-IR spectroscopy.

Please note that the indicator signals contained a significant amount of background, as can be seen in Figure 2 and Figure 3. Therefore, it is recommended to dilute the sample with fish oil used to prepare the calibration curve at a certain ratio to create a similar matrix environment when the calibration curve for analysis of other types of test samples is used. This can reduce the difference in the background signal. Additionally, the impact of the background was significantly reduced using the second-derivative method, as shown in Figure 3.

#### 3.2.2. Method Validation

The FT-IR method developed in this study was validated in terms of specificity, linearity, LOD, LOQ, accuracy, and precision based on the ICH guidelines. The detailed procedures for method validation were appropriately adjusted for FT-IR. First, the specificity was verified by confirming that the FT-IR spectrum pattern of USP krill oil reference material and those of raw materials and/or supplements were the same [28,36,40,41]. The specificity of the assay was demonstrated by comparing the spectrum of a 1:1 mixture of krill oil raw material and fish oil with those produced after adding PC and PE standards to that mixture. It was verified that when the PC standard was added, the PC indicator signal (I15) increased along with the total PL indicator signal (I11); whereas, when the PE standard was added, the total PL indicator signal (I11) increased, but the PC indicator signal remained unchanged (Figure S3).

The linearity of the proposed FT-IR method was confirmed through the correlation coefficient of calibration curves (R^2^ > 0.988) within the measurement ranges (Figure 3). This level of acceptability is similar to that of other quantitative analysis studies using FT-IR spectroscopy [42].

Table 2 lists the LOD and LOQ of the developed method for quantifying PC and total PL. The LOD and LOQ for the total PL assay by the second-derivative method were higher than the others due to the broadness of the indicator signal. However, considering the Codex standards that stipulate a total PL content of 30 wt% or more [9], the sensitivity of the proposed FT-IR method is suitable for determining the PC and total PL content of krill oil.

The recovery tests were performed in triplicate for three different content levels of PC and total PL to verify the accuracy of the proposed method. The 1:1 mixture sample (200 mg) was spiked with 100, 200, and 400 mg of the krill oil raw material. The average recovery results of PC and total PL using absorbance were 97.90% (96.34–99.70%) and 97.96% (95.45–101.07%), respectively. Relatively accurate results of 100.21% (98.58–101.94%) and 100.33% (96.42–104.18%) were obtained, respectively, when the second-derivative was used (see Table 2 and Tables S1 and S2 for details).

The precision of the developed FT-IR method was evaluated in terms of repeatability (intra-day) and intermediate precision (inter-day) expressed as the relative standard deviation (RSD). The RSDs of the repeatability and intermediate precision were 0.90–2.65% for PC and total PL assays (Table 2). Additionally, it was confirmed that the absolute SDs were at an appropriate level of 0.46–1.58 wt% (Tables S3 and S4). Therefore, the precision of the method was confirmed.

### 3.3. Application of Proposed FT-IR Method

The developed FT-IR method was applied to analyze 12 krill oil test samples: 5 mixture samples of krill oil raw material and fish oil with arbitrary ratios and 7 commercial supplement samples. The PC content and total PL content in the test samples analyzed through absorbance and second-derivative are summarized in Table 3 (Figure S4). Moreover, the analysis results by ^31^P NMR, an official method, are also presented for comparison.

The analysis of the mixture test samples showed very consistent results, with only a 0.20–1.38 wt% difference in the PC or total PL content compared to the ^31^P NMR results. This is because the krill oil raw material used to develop the FT-IR analysis method was also used in the newly prepared mixture test samples; however, it showed the consistency of the developed method.

The FT-IR analysis results of the supplement capsule samples in which different krill oil raw materials were used for production showed a difference of 0.21–6.21 wt% compared to the ^31^P NMR results, indicating a variation depending on the sample. Compared to the mixture test sample, the relatively large error in some supplement samples was likely due to the different profiles of PLs or other lipids or both in krill oil depending on the raw material, which affected the FT-IR spectrum. Characteristically, the average differences in content obtained by the second-derivative method in the supplement capsule samples were 0.81 wt% for PC and 1.83 wt% for total PL, which were smaller than the differences obtained when the absorbance method was used (3.09 and 2.25 wt% for PC and total PL, respectively). These results show that the second-derivative effectively reduces the influence of background signals according to the difference in raw materials.

As shown in Table 3, the total PL content in the krill oil supplements used in this study was analyzed at a level of 35–56 wt%, meeting the minimum 30 wt% specified in Codex. Additionally, the relative content of PC among total PL was at the level of 87–92% [34], which also meets the standard of 60–96% [33]. The application to the test samples confirmed that both FT-IR methods, that utilizing absorbance and that utilizing the second-derivative, can be easily applied to the analysis of krill oil samples having a PL content of approximately 20–56 wt%.

## 4. Conclusions

An FT-IR method using absorbance and the second-derivative was developed and validated for quantifying PC and total PL in krill oil samples. The signals of asymmetric stretch in –N–(CH_3_)_3_ (970 cm^−1^) and asymmetric phosphate diester stretch in PO_2_^−^ (1236 cm^−1^) were selected as indicator variables to determine PC and total PL content, respectively, and used to construct calibration curves. The developed FT-IR method was validated in terms of specificity, linearity, LOD, LOQ, accuracy, and precision according to the ICH guidelines. Furthermore, both the absorbance and second-derivative methods were confirmed to be suitable for PC and PL quantification. The developed FT-IR method was applied to the analysis of 12 test samples, and the results were found to agree with those obtained by the ^31^P NMR method within an average difference of 3 wt%. By validating the developed analytical method and confirming its applicability to general samples, it was confirmed that the FT-IR method can be used as a convenient and inexpensive alternative to ^31^P NMR techniques in analyzing the PL content of krill oil.

## Figures and Tables

**Table 1:** Assignment of absorption signals in FT-IR spectrum of krill oil reference sample [1,36,37].

Peak Variable	Wavenumber (cm^−1^)	Functional Group	Mode of Vibration
I1	3010	=C–H (*cis*-)	Stretching
I2	2958	–C–H (CH_3_)	Stretching (asym)
I3	2924	–C–H (CH_2_)	Stretching (asym)
I4	2853	–C–H (CH_2_)	Stretching (sym)
I5	1745	–C=O (ester)	Stretching
I6	1654	–C=C– (*cis-*)	Stretching
I7	1466	–C–H (CH_2_, CH_3_)	Bending (scissoring)
I8	1419	=C–H (*cis*-)	Bending (rocking)
I9	1395	=C–H (*cis*-)	Bending
I10	1377	–C–H (CH_3_)	Bending (sym)
I11	1236	PO_2_^−^ (diester)	Stretching (asym)
I12	1167	–C–O–P (ester)	Stretching (sym)
I13	1090	PO_2_^−^ (diester)	Stretching (sym)
I14	1060	–C–O–P (ester)	Stretching (asym)
I15	970	–N–(CH_3_)_3_	Stretching (asym)

**Table 2:** LOD, LOQ, recovery, repeatability, and intermediate precision of FT-IR method.

KERRYPNX	Absorbance		Second-Derivative	
	PC	Total PL	PC	Total PL
LOD (wt%)	1.19	0.65	0.35	3.29
LOQ (wt%)	3.59	1.98	1.06	9.98
Recovery (%)	97.90	97.96	100.21	100.33
Repeatability (%, RSD)	0.92	1.25	0.90	2.31
Intermediate precision (%, RSD)	1.47	0.93	1.15	2.65

**Table 3:** PC and total PL contents in krill oil test samples obtained from the FT-IR and ^31^P NMR methods.

Test Sample	PL Contents (wt%) ± Standard Deviation					
	FT-IR Method				^31^P NMR Method	
	Absorbance		Second-Derivative			
	PC	Total PL	PC	Total PL	PC	Total PL
Mixture 1	19.25 ± 0.31	22.00 ± 0.42	19.66 ± 0.06	22.89 ± 0.65	20.02 ± 0.02	22.68 ± 0.01
Mixture 2	22.44 ± 0.19	25.76 ± 0.10	23.14 ± 0.06	25.39 ± 0.81	22.04 ± 0.31	25.10 ± 0.24
Mixture 3	33.03 ± 0.56	37.92 ± 0.63	33.56 ± 0.28	37.89 ± 0.19	32.18 ± 1.08	36.56 ± 1.22
Mixture 4	36.10 ± 0.65	41.11 ± 0.72	36.83 ± 0.61	41.90 ± 0.41	36.71 ± 0.33	41.84 ± 0.33
Mixture 5	43.78 ± 0.44	49.99 ± 0.43	43.96 ± 0.03	50.54 ± 1.20	44.46 ± 0.11	50.74 ± 0.07
Supplement 1	50.95 ± 0.64	55.29 ± 0.69	50.01 ± 0.19	56.41 ± 1.29	49.39 ± 0.69	56.12 ± 0.78
Supplement 2	53.38 ± 0.37	54.30 ± 0.54	47.10 ± 0.69	52.58 ± 0.96	48.05 ± 1.53	52.14 ± 1.66
Supplement 3	35.89 ± 0.26	38.82 ± 1.03	34.95 ± 0.77	35.89 ± 1.17	35.21 ± 1.55	39.59 ± 1.74
Supplement 4	33.82 ± 1.35	38.39 ± 1.50	32.25 ± 0.77	34.12 ± 0.67	31.13 ± 1.85	35.50 ± 2.11
Supplement 5	37.94 ± 2.24	41.43 ± 2.12	34.94 ± 0.52	37.70 ± 0.37	34.06 ± 0.96	38.71 ± 1.09
Supplement 6	41.75 ± 0.58	42.64 ± 0.58	40.28 ± 0.77	44.33 ± 0.85	40.49 ± 0.10	45.23 ± 0.11
Supplement 7	44.94 ± 1.32	48.05 ± 1.27	37.13 ± 1.13	39.17 ± 0.79	38.73 ± 1.10	44.31 ± 1.26

## Data Availability

Not available.

## Supplementary Materials

The following are available online at https://www.mdpi.com/article/10.3390/foods11010041/s1, Figure S1: ^31^P NMR spectrum of USP standard of krill oil, Figure S2: ^31^P NMR spectrum of fish oil (PL free), Figure S3: Comparison of FT-IR spectra of a 1:1 mixture of krill oil raw material and fish oil with those produced after adding (a) PE and (b) PC standards to that mixture, Figure S4: Comparison of PC and total PL contents in krill oil test samples obtained from the FT-IR and ^31^P NMR methods, Table S1: Accuracy of the FT-IR method for PC with absorbance and second derivative, Table S2: Accuracy of the FT-IR method for total PL with absorbance and second derivative, Table S3: Repeatability of the FT-IR method for PC and total PL with absorbance and second derivative, Table S4: Intermediate precision of the FT-IR method for PC and total PL with absorbance and second derivative.
