# Supplementary material for: Development and Validation of a Simple Method to Quantify Contents of Phospholipids in Krill Oil by Fourier-Transform Infrared Spectroscopy
Source: Foods. 2021 Dec 24;11(1):41. doi: 10.3390/foods11010041 (PMC8750116; doi:10.3390/foods11010041)
Supplement: Supplementary file 1 [file foods-11-00041-s001.zip › foods-1503490-supplementary.pdf]

*foods*

MDPI

*Supplementary Materials*

# Development and Validation of a Simple Method to Quantify Contents of Phospholipids in Krill Oil by Fourier-Transform Infrared Spectroscopy

**Se-Eun Park [1,†], Hyo-Yeon Yu [1,†], and Sangdoo Ahn [1,*]**

[1] Department of Chemistry, Chung-Ang University, Seoul 06974, Republic of Korea; seeun6472@naver.com (S.P.), hyoyeonyu@gmail.com (H.Y.), sangdoo@cau.ac.kr (S.A.)
* Correspondence: sangdoo@cau.ac.kr (S.A.); Tel.: +82-2-820-5230 (S.A.)
† These authors contributed equally to this work.

**Citation:** Park, S.-E.; Yu, H.-Y.; Ahn, S. Development and Validation of a Simple Method to Quantify Contents of Phospholipids in Krill Oil by Fourier-Transform Infrared Spectroscopy. *Foods* **2022**, *11*, 41. https://doi.org/10.3390/foods11010041

Academic Editors: Annalisa De Girolamo and Vincenzo Lippolis

Received: 25 November 2021
Accepted: 21 December 2021
Published: 24 December 2021

**Publisher's Note:** MDPI stays neutral with regard to jurisdictional claims in published maps and institutional affiliations.

**Copyright:** © 2021 by the authors. Licensee MDPI, Basel, Switzerland. This article is an open access article distributed under the terms and conditions of the Creative Commons Attribution (CC BY) license (https://creativecommons.org/licenses/by/4.0/).

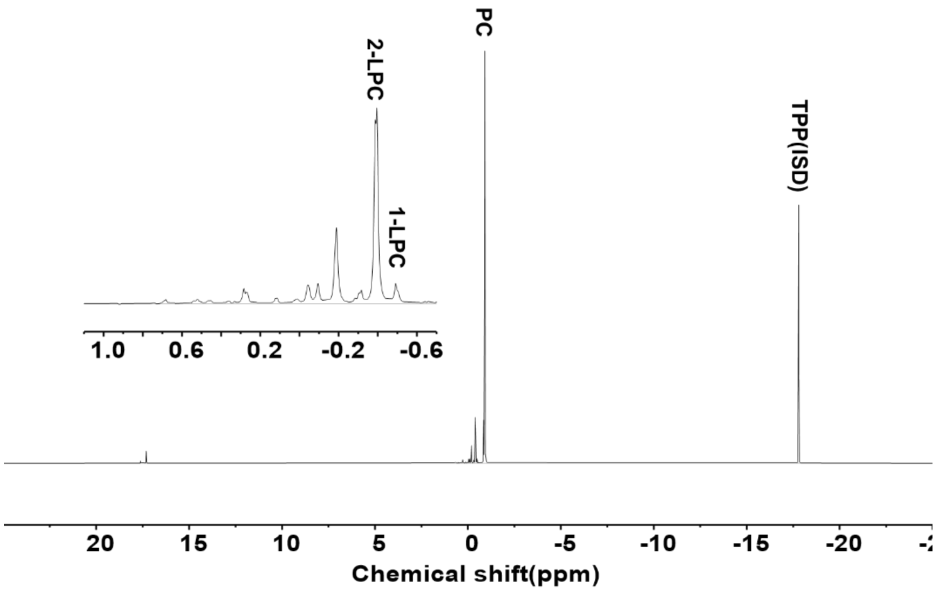


**Figure S1.** $^{31}P$ NMR spectrum of USP standard of krill oil.

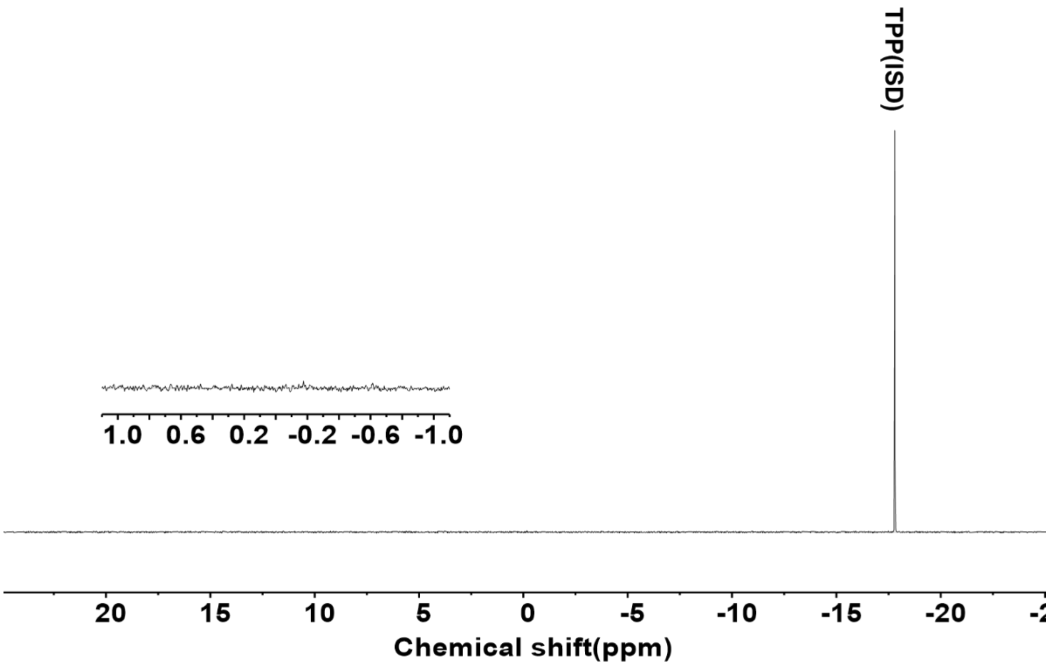


**Figure S2.** $^{31}P$ NMR spectrum of fish oil (PL free).

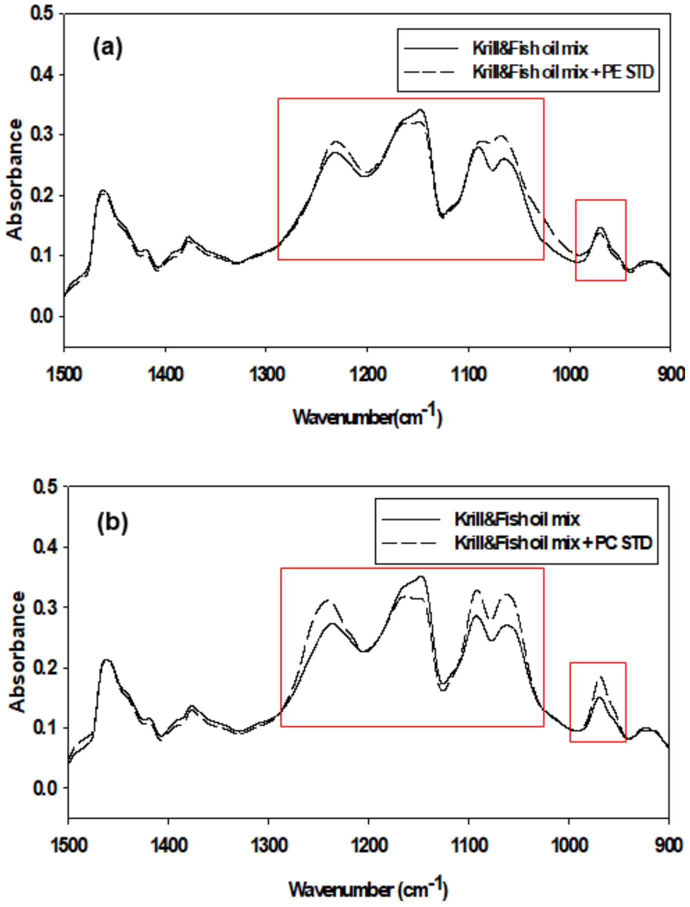


**Figure S3.** Comparison of FT-IR spectra of a 1:1 mixture of krill oil raw material and fish oil with those produced after adding (**a**) PE and (**b**) PC standards to that mixture.

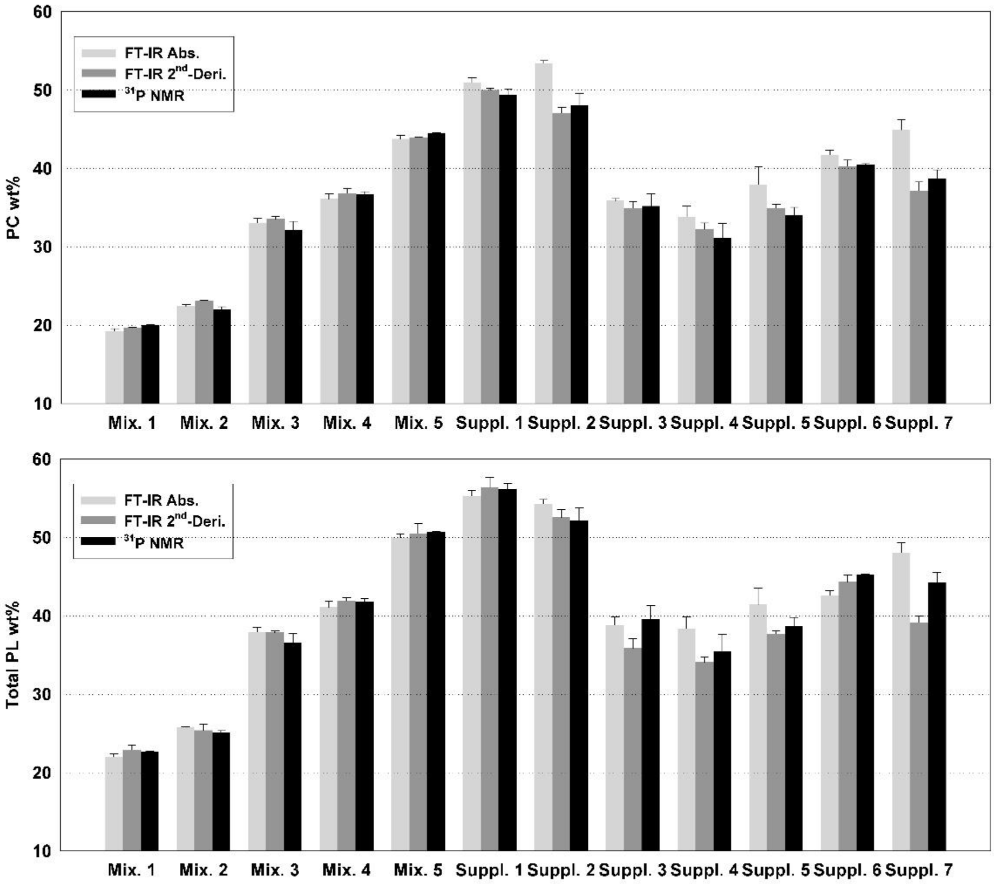


**Figure S4.** Comparison of PC and total PL contents in krill oil test samples obtained from the FT-IR and $^{31}P$ NMR methods.

**Table S1.** Accuracy of the FT-IR method for PC with absorbance and second derivative.

| No | Added (mg) | Absorbance | | Second Derivative | |
|---|---|---|---|---|---|
| | | Founded (mg) | Recovery (%) | Founded (mg) | Recovery (%) |
| 1 | 156.65 | 155.25 | 99.11 | 159.68 | 101.94 |
| 2 | 158.54 | 155.93 | 98.35 | 156.31 | 98.59 |
| 3 | 159.39 | 156.87 | 98.42 | 158.40 | 99.38 |
| 4 | 100.71 | 97.66 | 96.97 | 100.01 | 99.30 |
| 5 | 104.30 | 102.33 | 98.11 | 106.19 | 101.81 |
| 6 | 113.12 | 110.85 | 97.99 | 113.56 | 100.38 |
| 7 | 74.68 | 71.95 | 96.34 | 73.63 | 98.58 |
| 8 | 78.77 | 78.53 | 99.70 | 80.02 | 101.58 |
| 9 | 88.14 | 84.70 | 96.09 | 88.41 | 100.30 |
| Ave. | | | 97.90 | | 100.21 |

**Table S2.** Accuracy of the FT-IR method for total PL with absorbance and second derivative.

| No | Added (mg) | Absorbance | | Second Derivative | |
|---|---|---|---|---|---|
| | | Founded (mg) | Recovery (%) | Founded (mg) | Recovery (%) |
| 1 | 177.68 | 176.60 | 99.39 | 181.88 | 102.36 |
| 2 | 179.83 | 176.94 | 98.39 | 180.36 | 100.29 |
| 3 | 180.79 | 177.86 | 98.38 | 182.31 | 100.84 |
| 4 | 114.23 | 110.96 | 97.14 | 116.07 | 101.61 |
| 5 | 118.30 | 116.48 | 98.46 | 117.52 | 99.34 |
| 6 | 128.31 | 125.86 | 98.09 | 124.23 | 96.82 |
| 7 | 84.71 | 80.87 | 95.46 | 81.68 | 96.42 |
| 8 | 89.35 | 90.02 | 100.75 | 93.08 | 104.18 |
| 9 | 99.98 | 95.56 | 95.58 | 101.07 | 101.09 |
| Ave. | | | 97.96 | | 100.33 |

**Table S3.** Repeatability of the FT-IR method for PC and total PL with absorbance and second derivative.

| | Absorbance | | Second Derivative | |
|---|---|---|---|---|
| | PC (wt%) | Total PL (wt%) | PC (wt%) | Total PL (wt%) |
| 1 | 51.31 | 58.92 | 52.14 | 58.97 |
| 2 | 51.01 | 58.50 | 51.50 | 57.45 |
| 3 | 50.13 | 56.95 | 50.58 | 55.78 |
| 4 | 51.16 | 58.24 | 51.39 | 55.29 |
| 5 | 50.39 | 57.89 | 51.16 | 55.40 |
| 6 | 51.36 | 59.17 | 51.31 | 56.38 |
| Average (wt%) | 50.89 | 58.28 | 51.35 | 56.54 |
| SD (wt%) | 0.47 | 0.73 | 0.46 | 1.31 |
| RSD (%) | 0.92 | 1.25 | 0.90 | 2.31 |

**Table S4.** Intermediate precision of the FT-IR method for PC and total PL with absorbance and second derivative.

| | **Absorbance** | | **Second Derivative** | |
|---|---|---|---|---|
| | **PC (wt%)** | **Total PL (wt%)** | **PC (wt%)** | **Total PL (wt%)** |
| Day 1 | 49.88 | 57.04 | 49.31 | 58.57 |
| Day 2 | 51.66 | 58.13 | 50.54 | 58.12 |
| Day 3 | 51.16 | 58.23 | 50.53 | 61.67 |
| Average (wt%) | 50.90 | 57.80 | 50.13 | 59.45 |
| SD (wt%) | 0.75 | 0.54 | 0.58 | 1.58 |
| RSD (%) | 1.47 | 0.93 | 1.15 | 2.65 |
